# Epidemiology and inpatient treatment of vascular diseases in Germany: English Version

**DOI:** 10.1007/s00772-015-0095-5

**Published:** 2015-10-23

**Authors:** A. Kühnl, H. Söllner, H.-H. Eckstein

**Affiliations:** Klinik und Poliklinik für Vaskuläre und Endovaskuläre Chirurgie, Klinikum rechts der Isar, Technische Universität München, Ismaninger Straße 22, 81675 Munich, Germany

**Keywords:** Vascular epidemiology, Provision of vascular services, Administrative incidence, Vascular surgery, Vascular medicine, Vaskuläre Epidemiologie, Vaskuläre Versorgung, Krankenhausinzidenz, Gefäßchirurgie, Gefäßmedizin

## Abstract

**Background:**

Non-cardiac vascular diseases account for approximately 10 % of the total burden of disease in the German population as measured in disability-adjusted life years (DALY). Thus, from the social perspective, much attention should be paid to optimization of the provision of vascular services especially in elderly people.

**Objectives:**

This article describes the structure of inpatient care and the provision of vascular treatment as well as age- and gender-specific hospital incidence rates of vascular diseases in Germany between 2005 and 2013.

**Materials and methods:**

Secondary data analysis is based on basic data from German hospitals as well as nationwide aggregated data from the diagnosis-related groupsʼ statistics from the Federal Statistical Office.

**Results:**

Since 2005, the incidence of non-cardiac vascular diseases has increased and showed a significant dependency on sex and age. In general, men were affected consistently more frequently by vascular diseases. In contrast, hospital admission rates due to varicose veins or acute ischemia of the legs were higher in women. Treatment of arterial diseases was performed predominantly in general surgery units or departments for vascular surgery. From 1991 to 2013, the number of hospitals employing specialists for vascular surgery almost doubled, and the number of vascular surgeons working full-time in German hospitals was nearly tripled. Endovascular approaches were used particularly for revascularization of peripheral arteries as well as aortic aneurysm repair. In contrast, carotid surgery as well as peripheral embolectomy were predominately performed using open surgical techniques.

**Conclusions:**

Since 1991, the increasing need for vascular services for patients has been met by continuously growing structures for the provision of vascular surgical treatment; however, the suitability and efficiency of provision of vascular services could only be assessed in an appropriate way by using more differentiated and disaggregated data.

**Additional material available online:**

Additional information is available in the online version of this article (doi:10.1007/s00772-015-0095-5).

## Abstract (de)

**Hintergrund:**

In Deutschland sind ca. 10 % der Gesamtkrankheitslast (Burden of disease gemessen in DALY) auf nicht-kardiale Gefäßerkrankungen zurückzuführen. Aus gesamtgesellschaftlicher Perspektive ist der Versorgung von Patienten mit Gefäßerkrankungen somit große Bedeutung zuzumessen; insbesondere bei der älteren Bevölkerung.

**Ziel der Arbeit:**

Dieser Artikel stellt in deskriptiver Weise die stationären Versorgungsstrukturen sowie die alters- und geschlechtsspezifische Krankenhausinzidenz vaskulärer Erkrankungen und deren Versorgung in Deutschland von 2005 bis 2013 dar.

**Material und Methoden:**

Sekundärdatenanalyse basierend auf den Grunddaten der Krankenhäuser, der Diagnosedaten der Krankenhauspatientinnen und - patienten sowie der Fallpauschalenbezogenen Krankenhausstatistik (DRG-Statistik) des Deutschen Statistischen Bundesamtes.

**Ergebnisse:**

Gefäßerkrankungen nahmen zu und zeigten eine starke Alters- und Geschlechtsabhängigkeit**.** Männer waren durchwegs häufiger, bei Varizen der unteren Extremität sowie bei arteriellen Embolien oder Thrombosen in höheren Altersstufen jedoch seltener betroffen als Frauen. Die Versorgung arterieller Erkrankungen erfolgte überwiegende in Fachabteilungen für Gefäßchirurgie sowie ungeteilten Abteilungen für Chirurgie. Die Anzahl der Krankenhäuser mit ärztlichem Personal für Gefäßchirurgie hat sich zwischen 1991 und 2013 nahezu verdoppelt. Ebenso stieg die Anzahl der hauptamtlich in deutschen Krankenhäusern tätigen Gefäßchirurginnen und Gefäßchirurgen auf das fast Dreifache an. Endovaskuläre Verfahren dominierten eher bei peripheren Revaskularisationen und der Ausschaltung von Aortenaneurysmen, während Carotisrevaskularisationen und periphere Embolektomien weiterhin überwiegend offen-chirurgisch erfolgten.

**Schlussfolgerung:**

Zur Versorgung der zunehmenden Anzahl von Patienten mit Gefäßerkrankungen standen und stehen ebenso kontinuierlich wachsende gefäßchirurgische Versorgungsstrukturen zur Verfügung. Die Angemessenheit bzw. Bedarfsgerechtigkeit der Versorgung wäre jedoch nur mit differenzierteren, vor allem desaggregierten Daten genauer zu beurteilen.

## Introduction

In 2013, the leading causes of death in Germany were cardiovascular diseases (I00–I99), which accounted for approximately 40 % of deaths (*n* = 355,000) [1]. Diseases of the arteries (I70–I79) or veins (I80–I89) were documented as the cause of death in 23,900 deaths (2.4 % of all deceased). Compared with 1998, there were 8000 fewer deaths; however, the impact of improved medical treatment on the mortality of vascular diseases, demographic change, and the limited validity of the cause of death statistics [2] must be taken into consideration. If morbidity is considered on the basis of DALYs (disability-adjusted life years lost, see [3] for explanation), the Global Burden of Disease Study [4] showed that in Germany approximately 22 % of all DALYs (33 % for those in patients > 70 years old) were due to cardiovascular diseases in 2012. In non-cardiac vascular diseases, the values were 10 and 17 %, respectively. To reduce mortality and especially morbidity, care and treatment of vascular, non-cardiac diseases is also of great importance, particularly in the elderly population.

This article describes the structure of inpatient care as well as the age- and gender-specific hospital incidence rates of vascular diseases and their treatment in Germany in 2013.

## Methods

The number of departments of vascular surgery in German hospitals, the number of beds therein, the number of hospitals employing specialists in vascular surgery, and the number of full-time vascular surgeons were extracted from the basic data of German hospitals from the Federal Statistical Office ([5], as of 31 December 2013). The following definitions were used:

- Department: “Departments are organizationally demarcated units with treatment facilities that are typical for that specific specialty. The departments are constantly led by physicians. The department structure is adapted to the specialties of the physicians” [5].
- Full-time physicians: “This includes all the salaried physicians working in the institution. Consulting and visiting physicians are not included” [5].
- Chief physicians: “These are all full-time physicians working with a chief physician contract as well as those physicians who own licensed private clinics. Physicians with several specialties are classified according to the main activities that they perform” [5].
- Specialist physicians: “Physicians, insofar as they have completed further education, are classified according to their field of specialty” [5].

The data for the treating departments were extracted from the diagnosis data of the hospitalized patients ([6], as of 31 December 2013). The assignment of cases (not the patients) to the departments was already performed by the Federal Statistical Office and was based on the longest stay within a specific department.

The codings used in the analysis of the principal diagnoses and procedures in the hospitals were taken from the 2013 versions of the ICD-10 and the OPS catalog and are listed in Appendix 1 [7]. The information on hospital incidence rates, age and gender distribution of the main vascular diagnoses, and the vascular procedures performed at the hospitals are based on the DRG-related hospital statistics (DRG statistics) of the German Federal Statistical Office ([8], as of 31 December 2013).

Age standardization was based on the average population of 2013, according to the population census in Germany on the reporting dates 31 December 2012 and 31 December 2013 [9]. However, these tables did not provide separate information for the age groups 85–89, 90–95, and 95 + years. Therefore, in figures with age-standardized values, these three groups were summarized as the group “85 +.”

For the sake of direct comparability, alternative therapies (e.g., carotid endarterectomy vs. carotid stenting) are shown side by side and with the same scale on the y-axis, where appropriate.

## Results

### Hospitals and physicians in vascular surgery

**Fig. 1:**
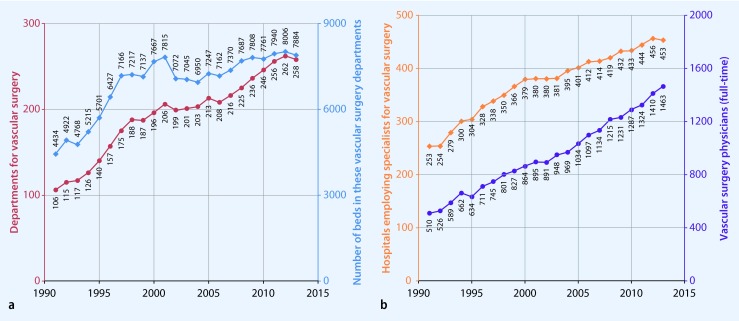
**a** Vascular surgery departments (*red*) and the number of beds therein (*blue*). **b** Hospitals employing specialists for vascular surgery (*orange*) and full-time specialists for vascular surgery (*violet*)

**Fig. 2:**
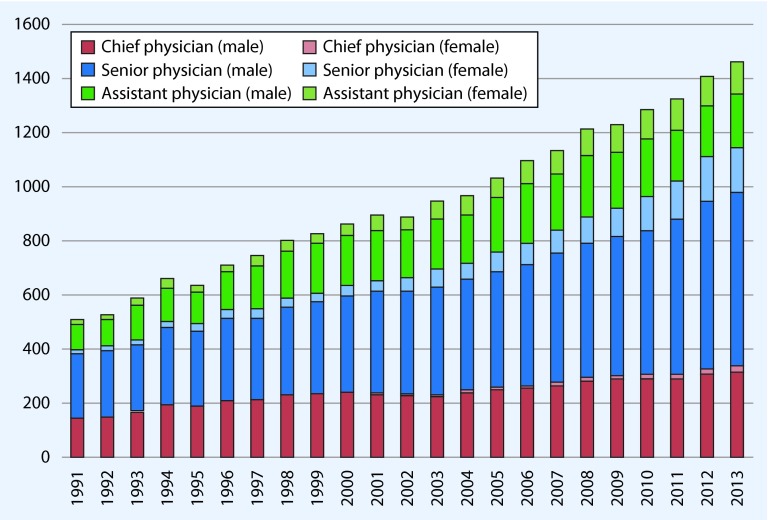
Full-time specialists employed in hospitals who have completed vascular surgery training (speciality, subspecialty) according to functional position and gender

Fig. 1a shows the number of vascular surgery departments registered by the Federal Statistical Office and the number of beds therein. In contrast to the rather rising numbers in recent years, the figures suggest a stagnation in both the number of beds and departments. Based on the number of beds, the average size of departments decreased between 1991 and 2013 from 42 to 31 beds. This is, however, based on arithmetic means, and it was not possible to provide the more appropriate *median* department size from the available data. The number of hospitals employing specialists in vascular surgery has almost doubled since 1991 (from 253 to 453). Likewise, the number of full-time physicians working in German hospitals (male and female individuals) who had completed further education in vascular surgery rose almost threefold from 510 to 1463 (Fig. 1b). Since 1991, the number of (male) physicians for vascular surgery (chief, senior, and assistant physicians) slightly more than doubled (from 478 to 1156), while the number of female chief physicians rose 11-fold (from 2 to 22); the number of female senior physicians rose 13-fold (from 13 to 166); and the number of female assistant physicians rose 7-fold (from 17 to 119). This change over time is illustrated in Fig. 2.

### Treating departments

**Fig. 3:**
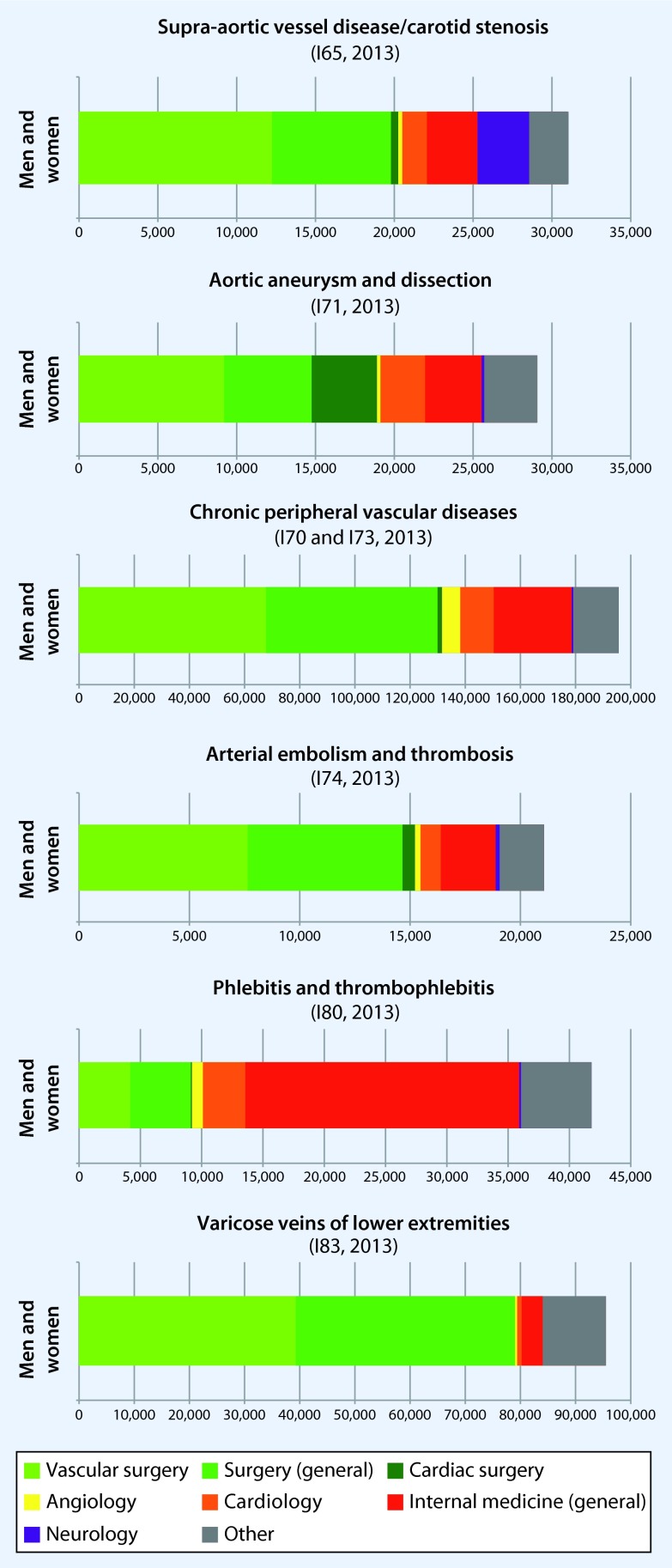
Number of departments in 2013. All data are solely available based on the three-digit ICD-10 code level

Approximately two-thirds of supra-aortic vascular diseases (I65, primarily stenosis of the internal carotid artery) were treated in departments of vascular surgery or in general surgery or cardiac units, as well as 5–10 % each were treated in cardiology, internal medicine, or neurology units (Fig. 3). A similar scenario was seen in chronic (I70, I73) and acute (I74) peripheral vascular disorders, of which approximately 65 and 70 %, respectively were treated by (vascular) surgery departments and approximately 15–20 % by cardiology or general internal medicine units. No clear differences between genders were observed. However, the situation was different for aneurysms or aortic dissections, where 13 of men and 19 % of women were treated in cardiac surgery departments. These differences are most likely due to the fact that the relative proportion of dissections in the overall diagnosis group I71 was higher in women than in men. A differential analysis by dissections (I71.0) and aneurysms (I71.1–9) and by department was not possible because only pooled data were available.

Varicose veins of the lower extremities were treated in over 80 % of cases in (vascular) surgery departments, while in approximately 60 % of cases, the treatment of venous thrombosis and (thrombo)phlebitis was performed in departments for cardiology and general internal medicine units.

### Vascular principal diagnoses—overview and changes over time

**Fig. 4:**
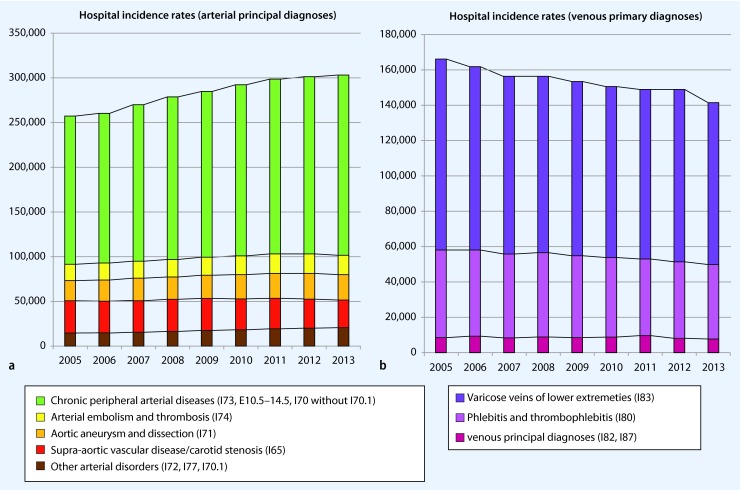
Cases in German hospitals with arterial or venous hospital principal diagnoses from 2005 to 2013

Since 2005, there has been a continuous increase in the hospital incidence rate of arterial principal diagnoses (+ 18 %), mainly due to an increase in chronic peripheral arterial disorders, aneurysms, and dissections (+ 47,000 cases since 2005; Fig. 4a). By contrast, there was a drop in the hospital incidence rate of supra-aortic vascular diseases (I65) by approximately 4,600 cases (− 13 %) from 2005 to 2013. In parallel, the number of venous principal diagnoses decreased by 24,000 cases (− 15 %) since 2005. However, especially for the venous diagnoses, it should be noted that only the principal diagnoses at hospitals were recorded here, and it should be assumed that a significant portion of treatments were provided by the private practice sector (Fig. 4b).

### Vascular principal diagnoses—age- and sex-specific hospital incidence rates

**Fig. 5:**
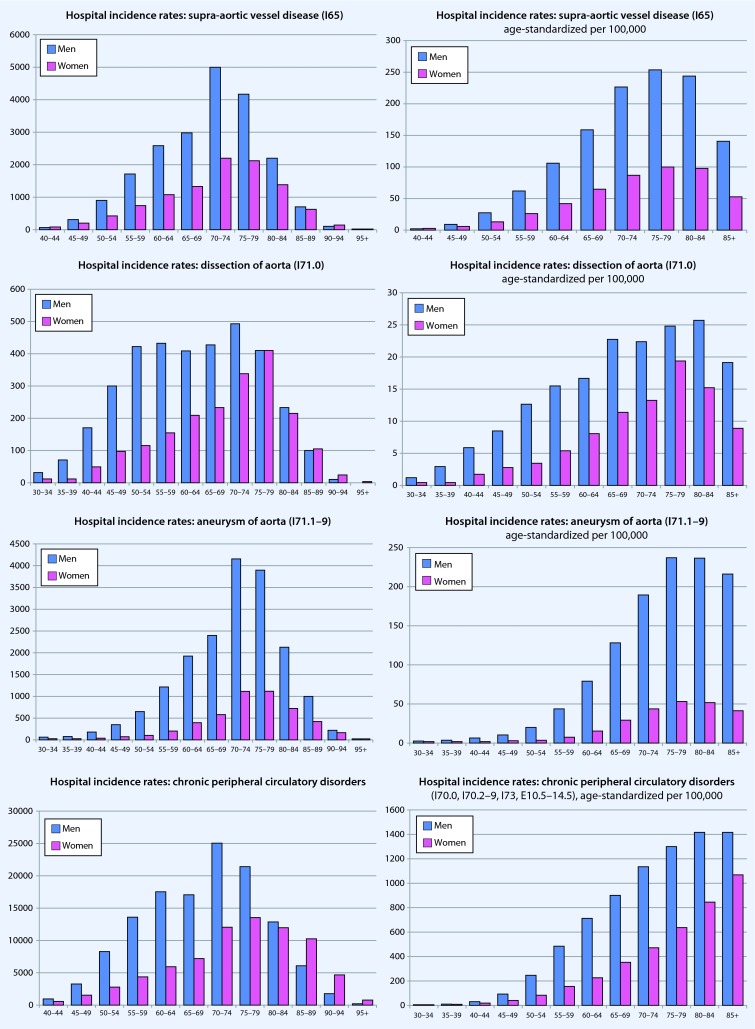

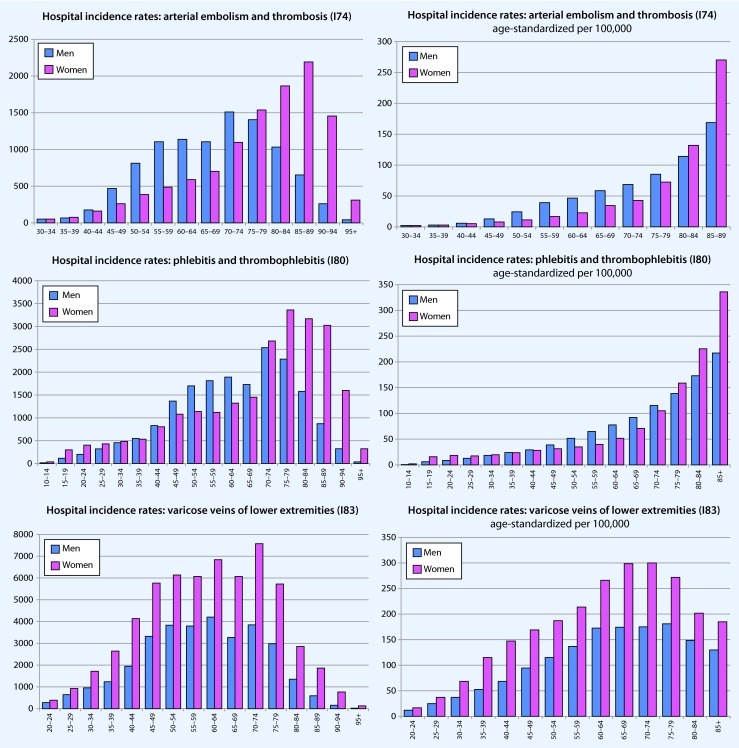
Age- and sex-specific presentation of hospital incidence rates of arterial and venous diseases in Germany in 2013. *Left* absolute case numbers, *right* age-standardized hospital incidence rates per 100,000 population (*blue* = men, *pink* = women)

In addition to the age-standardized presentation (Fig. 5, right column, respectively per 100,000 persons in each age category), the hospital incidence rates are provided as raw data (Fig. 5, left column) to reflect the real world situation and to provide an approximate estimate of the absolute burden of diseases and, thus, treatment needs. The absolute numbers shows that, in general, more men than women are hospitalized due to arterial disease. Furthermore, disease was clearly dependent on age. The distribution is similar in women and men; however, in women, there was the tendency for the peak incidence to be shifted to higher ages for (1) arterial thrombosis/embolism, (2) chronic peripheral arterial disorders, and (3) venous thromboses/(thrombo)phlebitis. Only for the principal diagnosis, “varicose veins of lower extremities” were higher in women than those for men treated — regardless of age — on an inpatient basis. The age-standardized values are listed in the right column of Fig. 5.

### Selected treatment procedures

**Fig. 6.**
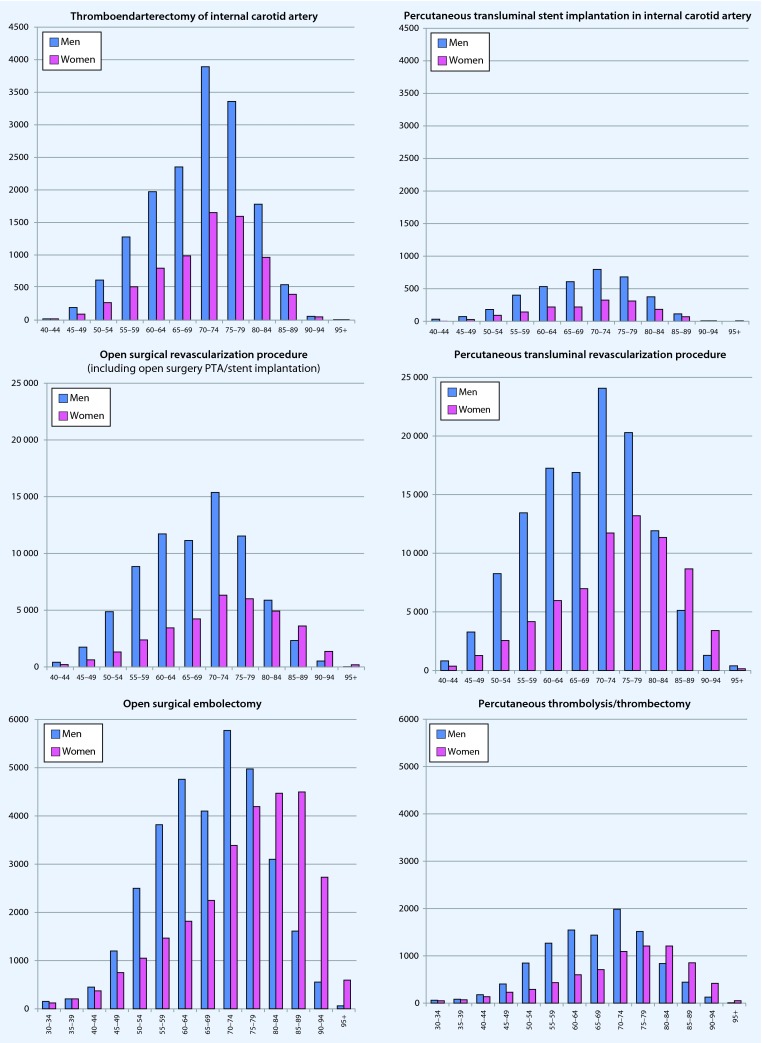

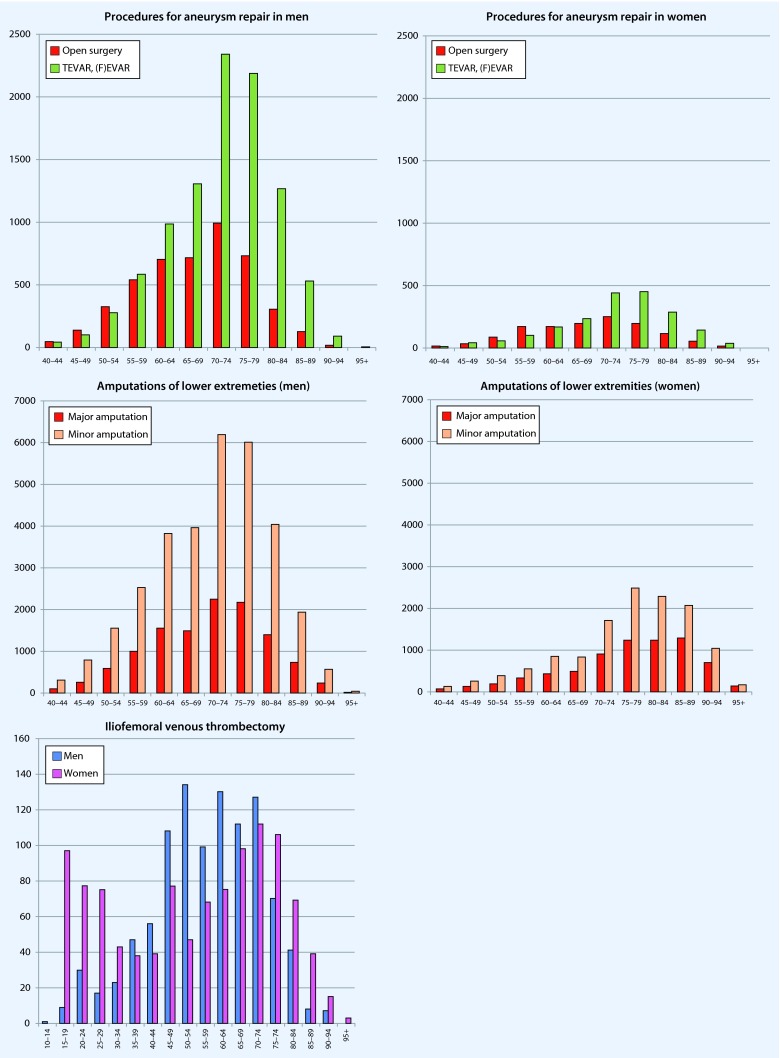
Age- and sex-specific presentation of procedures performed in Germany in 2013. Age-standardized presentation of the data was omitted (see text). When not otherwise stated: *blue* = men, *pink* = women

In general, most procedures were performed on patients between 70 and 75 years; women tended to be somewhat older (Fig. 6). In the treatment of supra-aortic vascular diseases, the number of carotid endarterectomies (CEA) compared with endovascular procedures (CAS) was considerably higher. In peripheral arterial disorders, the trend to perform open surgical peripheral revascularization techniques was less often than percutaneous transluminal revascularization procedures. By contrast, open surgical embolectomy was performed significantly more often than percutaneous embolectomy or lyses. Due to the significant overlap of the indications, possible multiple interventions; the lack of case designation for the respective procedures; and the fact that percutaneous transluminal revascularization can also be used in acute limb ischemia, no further reliable conclusions can be drawn from the available (pooled) data.

Open surgical aortic aneurysm repair, which was reported to be the trend in recent years [10], was performed less often than endovascular techniques, which in absolute terms were primarily used in elderly patients.

Overall, more amputations were performed in men than in women; however, the amputations were mostly minor—in both sexes and all age groups.

A venous thrombectomy was increasingly performed in both men and women with increasing age, but with approximately the same frequency. The exception was the group of women between 15 and 30 years, in whom a venous thrombectomy was performed significantly more often than in men of the same age; however, there was a very small number of total cases.

## Limitations

All presentations and analyses are based on routine (administrative) or secondary data. Regarding the usual limitations of this database, the reader is referred to Swart et al. [11]. When considering Figs. 1 and 2, there were consistently approximately 30 % more chief physicians than departments of vascular surgery. However, according to definition, these physicians had to have a chief physician contract and to work primarily in vascular surgery. In addition, there were approximately twice as many clinics employing specialists for vascular surgery compared with departments for vascular surgery. It is therefore not unlikely that the number of departments (in accordance with [5]) underestimates the real number of departments and department-like organisational constructs for vascular surgery. This is directly associated with the number of vascular surgery beds. First, only beds reported by the aforementioned departments were counted, and second, it was not known how many shared beds (e.g., visceral and vascular surgery) were reported to the Federal Statistical Office. Thus, it is also likely that the actual number of vascular surgery beds available is underestimated.

As shown in the individual graphs of Figs. 5 and 6, only the total number of coded hospital principal diagnoses and procedures are provided, but not the number of patients treated or cases. As reference to case or patient is lacking due to the structure of the data, a standardization of procedures by age or number of inhabitants would not have been appropriate. Since it can be assumed that vascular surgery procedures can also include more than a single procedure, the number of operations per patient is per se likely to be overestimated. Therefore, it was only possible to implicitly estimate a diagnosis- or indication-specific allocation. For further limitations of this database, the reader is referred to Eckstein et al. [12].

## Conclusion

The specialty of vascular surgery has continued to grow over the past 20 years. This is demonstrated by the steady increase in the use of vascular surgery services, the increase in the number of vascular surgery departments and clinics employing specialists in vascular surgery as well as by the increasing number of newly qualified specialists, and the increasing number of specialists working in vascular surgery, fortunately also as senior and chief physicians [10, 13]. The treatment of arterial disease and varicose veins is predominantly performed in specialized departments of vascular surgery or general surgery units. Only the treatment of thrombosis and dissections were increasingly performed in general internal medicine or cardiac surgery units.

The hospital incidence rates of vascular diseases, especially arterial diseases, continues to increase and is strongly dependent on age and sex, so that a differentiated evaluation should always take into account the age structure and sex distribution of the population. Men were consistently treated more often for vascular diseases, while women were more often affected by varicose veins of the lower extremities and arterial embolism or thrombosis.

Endovascular procedures tend to dominate in peripheral revascularization and treatment of aortic aneurysms in older patients, while carotid revascularization and peripheral embolectomy continue to be predominantly performed using open surgery procedures.

## Electronic supplementary material

(DOCX 17 kb)
