# Supplementary material for: Epidemiology and inpatient treatment of vascular diseases in Germany: English Version
Source: Gefasschirurgie. 2015 Oct 23;21(Suppl 1):14–23. doi: 10.1007/s00772-015-0095-5 (PMC4767870; doi:10.1007/s00772-015-0095-5)
Supplement: Supplementary file 1 — (DOCX 17 kb) [file 772_2015_95_MOESM1_ESM.docx]

| **Appendix 1:** Zuordnung der verwendeten OPS-Codes. | | | |
| --- | --- | --- | --- |
| **Thromendarteriektomie A.car.int.** | 5-381.02 |  |  |
| **Perkutane transluminale Stentimplantation (max. 2 Stents) A.car.int.** | 8-840.0k  8-840.0m  8-840.0k  8-840.0m | 8-840.1k  8-840.1m  8-840.1k  8-840.1m |  |
| **Offen-chirurgische, periphere Revaskularisationsverfahren inkl. offen-chirurgischer PTA/Stenteinlage** | 5-381.33  5-381.52  5-381.53  5-381.54  5-381.70  5-381.71  5-381.72  5-393.13  5-393.14  5-393.17  5-393.18  5-393.32  5-393.33  5-393.35  5-393.36  5-393.38  5-393.3x | 5-393.41  5-393.42  5-393.43  5-393.44  5-393.45  5-393.46  5-393.47  5-393.51  5-393.52  5-393.53  5-393.54  5-393.55  5-393.56  5-393.57  5-393.61  5-393.62 | 5-38e.01  5-38e.02  5-38e.03  5-38e.09  5-38e.0b  5-38e.0c  5-38e.0e  5-38e.11  5-38e.12  5-38e.13  5-38e.19  5-38e.1b  5-38e.1c  5-38e.1e  5-38f.1  5-38f.2  5-38f.3  5-38f.9  5-38f.b  5-38f.c  5-38f.e |
| **Perkutane-transluminale, periphere Revaskularisationsverfahren** | 8-836.04  8-836.09  8-836.0b  8-836.0c  8-836.0e  8-840.04  8-840.14  8-840.24  8-840.34  8-840.44  8-840.09  8-840.19  8-840.29  8-840.39  8-840.49 | 8-840.0b  8-840.1b  8-840.2b  8-840.3b  8-840.4b  8-840.5b  8-840.0c  8-840.1c  8-840.2c  8-840.3c  8-840.0e  8-840.1e  8-840.2e  8-840.3e |  |
| **Offen-chirurgische Embolektomie** | 5-380.00  5-380.01  5-380.02  5-380.03  5-380.04  5-380.05  5-380.06  5-380.0x  5-380.11  5-380.12  5-380.13  5-380.1x  5-380.20  5-380.21  5-380.22  5-380.23  5-380.24  5-380.25  5-380.26  5-380.27  5-380.28  5-380.2x | 5-380.30  5-380.31  5-380.32  5-380.33  5-380.34  5-380.35  5-380.3x  5-380.40  5-380.41  5-380.42  5-380.43  5-380.4x  5-380.51  5-380.52  5-380.53  5-380.54  5-380.55  5-380.56  5-380.5x | 5-380.60  5-380.61  5-380.62  5-380.63  5-380.64  5-380.65  5-380.66  5-380.67  5-380.6x  5-380.70  5-380.71  5-380.72  5-380.73  5-380.7x  5-380.80  5-380.81  5-380.82  5-380.83  5-380.84  5-380.85  5-380.86  5-380.87  5-380.8x |
| **Perkutane Thrombolyse/Thrombektomie** | 8-836.71  8-836.72  8-836.73  8-836.79  8-836.7a  8-836.7b  8-836.7c  8-836.7e | 8-836.81  8-836.82  8-836.83  8-836.89  8-836.8a  8-836.8b  8-836.8c  8-836.8e |  |
| **Amputationen** | **(major)**  5-864.0  5-864.1  5-864.2  5-864.3  5-864.4  5-864.5  5-864.6  5-864.7  5-864.8  5-864.9  5-864.a  5-864.x  5-864.y  5-865.0  5-865.1  5-865.2  5-865.3 | **(minor)**  5-865.4  5-865.5  5-865.6  5-865.7  5-865.8  5-865.x  5-865.y |  |
| **Offen-chirurgische Aneurysmaausschaltung (ohne Aorta ascendens und Aortenbogen)** | 5-384.31  5-384.32  5-384.3x  5-384.41  5-384.42  5-384.43  5-384.44  5-384.45  5-384.46  5-384.4x | 5-384.51  5-384.52  5-384.53  5-384.54  5-384.55  5-384.56  5-384.5x  5-384.61  5-384.62  5-384.63  5-384.64  5-384.65  5-384.66  5-384.6x | 5-384.71  5-384.72  5-384.73  5-384.74  5-384.75  5-384.76  5-384.7x |
| **Endovaskuläre Aneurysmaausschaltung (ohne Aorta ascendens und Aortenbogen)** | 5-38a.0  5-38a.12  5-38a.13  5-38a.14  5-38a.16  5-38a.17  5-38a.18  5-38a.19  5-38a.1a  5-38a.1b  5-38a.1c  5-38a.1d  5-38a.1e  5-38a.1f  5-38a.1g  5-38a.1h  5-38a.1j  5-38a.1k  5-38a.1m  5-38a.1n  5-38a.1p  5-38a.1q  5-38a.1r  5-38a.1s  5-38a.1v  5-38a.1x | 5-38a.70  5-38a.72  5-38a.73  5-38a.74  5-38a.75  5-38a.76  5-38a.77  5-38a.78  5-38a.7a  5-38a.80  5-38a.81  5-38a.82  5-38a.83  5-38a.84  5-38a.85  5-38a.86  5-38a.87  5-38a.88  5-38a.89  5-38a.8a  5-38a.8b |  |
| **Operative (OP) und endovenöse (EVLT/RFO) Varizentherapie** | **(OP)**  5-385.70  5-385.72  5-385.74  5-385.80  5-385.82  5-385.84  5-385.90  5-385.92  5-385.94  5-385.96  5-385.9x | **(EVLT)**  5-385.a1 | **(RFO)**  5-385.b |
| **Offen-chirurgische venöse Thrombektomie der Becken-Bein-Strombahn** | 5-380.97  5-380.98  5-380.99  5-380.9a  5-380.9b  5-380.9c |  |  |
| **Endovenöse Lyse** | keine spezifischen ICD-10 Codes verfügbar |  |  |
